# Evaluating the Efficacy of Soothing Agents in Mitigating 0.1% Retinol‐Induced Skin Irritation: A Patch Test

**DOI:** 10.1111/jocd.70488

**Published:** 2025-10-03

**Authors:** Ye Ying, Ye Fang, Xu Chenlan, Sun Lin, Huang Jiefang, Li Yanan, Sun Peiwen

**Affiliations:** ^1^ Research & Innovation Center Proya Cosmetics Co., Ltd. Hangzhou China

**Keywords:** clinician erythema assessment, patch test, retinol, skin barrier repair, soothing agents

To the Editor,

1

Retinol, a derivative of vitamin A, is a cornerstone in dermatology, widely used for its proven efficacy in promoting collagen synthesis, improving skin texture, and reducing fine lines, acne, and pigmentation. These benefits stem from retinol's ability to stimulate fibroblasts and reduce collagen‐degrading enzymes. However, retinol also induces skin irritation in many individuals, including erythema, stinging, and desquamation, which occurs through the activation of nuclear retinoic acid receptors and cytokine release. This irritation presents a challenge in dermatology and cosmetic formulations, as it limits retinol's applicability for sensitive skin. Thus, strategies to mitigate irritation have become a focal point in both clinical and cosmetic research. However, few clinical studies systematically comparing the soothing capabilities of representative soothing agents have been reported.

In this study, a randomized, double‐blind, vehicle‐controlled 5‐day patch test was conducted to map and rank the soothing and barrier‐enhancing efficacy of 15 potential treatments (Table 1), all claiming moisturizing, soothing, barrier‐enhancing, or anti‐inflammatory properties. A 0.1% retinol serum was used as the vehicle, which aligns with established usage for the Chinese population due to its balance between efficacy and irritation potential [1]. The patch test involved 21 healthy Chinese subjects, aged between 21 and 35 years, and was carried out using a similar 5‐day patch test method as reported in our previous article [2].

**TABLE 1:** Treatment, mean cumulative irritancy index (MCII ± SD), and cumulative Grade 2(+) reaction count for 0.1% retinol combined with soothing agents (6 time points summed).

Treatment	MCII, mean ± SD	Cumulative grade 2(+) count
*Good benefit (no Grade 2 reactions)*		
0.1% Retinol+2% PLG	0.26 ± 0.24*	0
0.1% Retinol+5% *Ceramides*	0.42 ± 0.31*	0
0.1% Retinol+3% Acetyl glucosamine	0.51 ± 0.32*	0
0.1% Retinol+2% Panthenol	0.55 ± 0.35*	0
0.1% Retinol+0.2% *TECA*	0.62 ± 0.30*	0
0.1% Retinol+0.5% *Centella asiatica*	0.68 ± 0.25^∆^	0
*Moderate benefit (Grade 2 reactions; significant or trend vs. vehicle)*		
0.1% Retinol+0.8% PLG	0.50 ± 0.36*	4
0.1% Retinol+5% *Calmsoon*	0.63 ± 0.34*	2
0.1% Retinol+5% *Collagen*	0.64 ± 0.36^∆^	4
0.1% Retinol+0.5% Ectoin	0.66 ± 0.40^∆^	2
0.1% Retinol+0.5% *SymCalmin*	0.66 ± 0.35^∆^	4
0.1% Retinol+0.1% Carboxymethyl Chitosan	0.67 ± 0.25^∆^	1
*No soothing benefit (Grade 2 reactions; no significant difference vs. vehicle)*		
0.1% Retinol+4% Sens' flower SD‐SC	0.76 ± 0.31	4
0.1% Retinol+0.4% *Crocus sativus* extract	0.78 ± 0.29	3
0.1% Retinol+0.2% Bisabolol	0.78 ± 0.29	5
0.1% Retinol (vehicle)	0.77 ± 0.24	1
*Blank*	0.12 ± 0.15*	0

*Note:* Treatments are grouped by benefit category: Good benefit (no Grade 2 reactions), Moderate benefit (Grade 2 reactions; significant or trend vs. vehicle), and No soothing benefit (Grade 2 reactions; no significant difference vs. vehicle). *p*‐value annotations versus vehicle are shown (*p < 0.05; Δ 0.05 ≤ *p* < 0.10).

According to the Clinical Erythema Assessment (CEA), compared to the 0.1% retinol (vehicle), the 15 treatments were divided into three categories: good benefit, moderate benefit, and no soothing benefit. Treatments in the good benefit category showed significant or directionally significant differences from the 0.1% retinol (vehicle) without any grade 2 (+) reactions. The moderate benefit category showed significant or directionally significant differences from the 0.1% retinol (vehicle) with grade 2 (+) reactions. The no soothing benefit category showed no significant differences from the 0.1% retinol (vehicle) with grade 2 (+) reactions. The two patch tests mainly yielded 0 (−) no reaction and 1 (±) doubtful reaction; however, after the second patch test, some grade 2 (+) reactions emerged. Interestingly, the 0.1% Retinol +2% PLG treatment had the lowest mean cumulative irritation index (MCII = 0.26) among all 15 treatments, reaffirming its strong skin‐soothing effects and correlating with our previous study [2]. Additionally, the 2% concentration appeared more effective than the 0.8% concentration, suggesting a possible dose‐dependent effect. The most pronounced erythema reaction was detected 24 h following the second patch removal, with representative Visia‐CR images depicted in Figure 1, and full time‐course counts are provided in Table S1.

**FIGURE 1:**
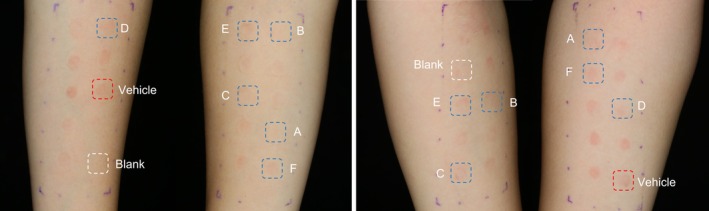
Visia‐CR images indicating the erythema response at 24 h after the second patch removal. (A) 0.1% retinol + 2% PLG. (B) 0.1% retinol + 5% ceramides; (C) 0.1% retinol + 3% acetyl glucosamine; (D) 0.1% retinol + 2% panthenol; (E) 0.1% retinol + 0.2% TECA; (F) 0.1% retinol + 0.5% *Centella asiatica* . Left, subject 011, female, 31 years old; Right, subject 023, female, 25 years old.

The mechanisms underlying retinol‐induced irritation are complex, involving disruption of the skin's lipid barrier, increased transepidermal water loss, and heightened inflammation. Therefore, incorporating ingredients that support barrier repair and reduce inflammation is essential. PLG and ceramides, for instance, play a critical role in restoring the skin's lipid barrier, while acetyl glucosamine, panthenol, and *Centella asiatica* support skin regeneration and anti‐inflammatory responses, improving skin resilience to retinol [2, 3, 4, 5, 6].

This study found that 0.1% retinol combined with 2% PLG, 5% ceramides, 3% acetyl glucosamine, 2% panthenol, 0.2% TECA, and 0.5% *Centella asiatica* was the most effective treatment for reducing retinol‐induced irritation and promoting skin barrier repair. These findings offer a practical approach for formulating retinol‐based products suitable for sensitive skin, broadening retinol's applicability, and ensuring its safe use. Further investigation into the synergistic effects of these treatments and validation of their clinical efficacy in larger skincare trials would be valuable. To the best of our knowledge, this study is the first to allow a comparison between reference soothing and barrier‐enhancing ingredients for retinol‐induced skin irritation and will help clinicians propose the most effective approach for their patients.

## Author Contributions

Ye Ying designed the research study and performed the research. Ye Fang analyzed the data and wrote the original draft. Xu Chenlan, Sun Lin, and Huang Jiefang performed the research and collected the data. Li Yanan designed the research study and revised the draft. Sun Peiwen supervised the research and revised the draft. All authors read and approved the final manuscript.

## Ethics Statement

The protocol of the patch test study (PCS‐HPT‐22010) had passed review by the Ethical Commission of Proya Cosmetics Co., Ltd. The subjects in this manuscript have given written informed consent to the publication of their case details.

## Consent

All participants granted permission for the dissemination of their photographs and medical data by the authors during the submission process. They also consented to the potential publication of their photographs and medical details both in print and online, acknowledging the possibility of public access.

## Conflicts of Interest

The authors declare no conflicts of interest.

## Supporting information


**Table S1:** Number of erythema reactions recorded (Grade 1 + Grade 2) at six post‐removal observation timepoints (after the 1st 48‐h patch: 0.5, 24 h; after the 2nd 72‐h patch: 0.5, 24, 48, 96 h) across all treatments.

## Data Availability

The data that support the findings of this study are available from the corresponding author upon reasonable request.
