# Supplementary material for: Evaluating the Efficacy of Soothing Agents in Mitigating 0.1% Retinol‐Induced Skin Irritation: A Patch Test
Source: J Cosmet Dermatol. 2025 Oct 3;24(10):e70488. doi: 10.1111/jocd.70488 (PMC12494960; doi:10.1111/jocd.70488)
Supplement: Supplementary file 1 — Table S1: Number of erythema reactions recorded (Grade 1 + Grade 2) at six post‐removal observation timepoints (after the 1st 48‐h patch: 0.5, 24 h; after the 2nd 72‐h patch: 0.5, 24, 48, 96 h) across all treatments. [file JOCD-24-e70488-s001.docx]

**Supporting information**

Table S1. Number of erythema reactions recorded (Grade 1 + Grade 2) at six post-removal observation timepoints (after the 1st 48-h patch: 0.5h, 24h; after the 2nd 72-h patch: 0.5h, 24h, 48 h, 96h) across all treatments.

| **Treatment** | **1^st^0.5h** | **1^st^24h** | **2^nd^0.5h** | **2^nd^24h** | **2^nd^48h** | **2^nd^96h** |
| --- | --- | --- | --- | --- | --- | --- |
| **Good benefit (no Grade 2 reactions)** | | | | | | |
| 0.1% Retinol+2% PLG | 4 | 3 | 9 | 11 | 4 | 2 |
| 0.1% Retinol+5% *Ceramides* | 9 | 9 | 10 | 11 | 8 | 6 |
| 0.1% Retinol+3% Acetyl glucosamine | 9 | 10 | 15 | 13 | 9 | 8 |
| 0.1% Retinol+2% Panthenol | 7 | 9 | 12 | 14 | 14 | 13 |
| 0.1% Retinol+0.2% *TECA* | 10 | 7 | 18 | 17 | 12 | 14 |
| 0.1% Retinol+0.5% *Centella asiatica* | 10 | 11 | 17 | 19 | 15 | 14 |
| **Moderate benefit (Grade 2 reactions; significant or trend vs vehicle)** | | | | | | |
| 0.1% Retinol+0.8% PLG | 8 | 6 | 12 | 10+3 | 8+1 | 11 |
| 0.1% Retinol+5% *Calmsoon* | 10 | 11 | 15 | 14+1 | 13+1 | 12 |
| 0.1% Retinol+5% *Collagen* | 11 | 9 | 16 | 13+3 | 13+1 | 11 |
| 0.1% Retinol+0.5% Ectoin | 12 | 9 | 17 | 14+1 | 14+1 | 13 |
| 0.1% Retinol+0.5% *SymCalmin* | 10 | 8 | 17 | 14+3 | 13+1 | 13 |
| 0.1% Retinol+0.1% Carboxymethyl Chitosan | 11 | 6 | 14 | 18+1 | 17 | 16 |
| **No soothing benefit (Grade 2 reactions; no significant difference vs vehicle)** | | | | | | |
| 0.1% Retinol+4% Sens’flower SD-SC | 13 | 11 | 18 | 17+2 | 14+2 | 15 |
| 0.1% Retinol+0.4% Crocus sativus extract | 10 | 12 | 17 | 18+1 | 17+2 | 18 |
| 0.1% Retinol+0.2% Bisabolol | 12 | 10 | 18 | 16+3 | 16+2 | 16 |
| 0.1% Retinol (vehicle) | 13 | 13 | 19 | 17 | 16+1 | 17 |
| Blank | 4 | 0 | 8 | 2 | 1 | 0 |

Footnotes (INCI full names):

PLG: Phytosteryl/Octyldodecyl Lauroyl Glutamate.

*Ceramides*: Glycerin, Hexyldecanol, Water, Hydrogenated Lecithin, Ceramide NP, Cholesterol, Perilla Ocymoides Seed Oil, Ceramide NS/Ceramide NG, Ceramide AP, Salicyloyl Phytosphingosine, Phytosphingosine, Tricetyl Phosphate.

*TECA*: Asiaticoside, Madecassic Acid, Asiatic Acid.

*Centella asiatica*: Madecassoside, Centella Asiatica Extract, Asiaticoside.

*Calmsoon*: Water, Glycerin, 1,2-Hexanediol, Hydroxyacetophenone, Beta-Glucan, Xanthan Gum.

*Collagen:* Water, Propanediol, Collagen.

*Symcalmin*: Pentylene Glycol, Butylene Glycol, Hydroxyphenyl Propamidobenzoic Acid, Ascorbyl Palmitate.

*Sens’flower SD-SC*: Water, Propanediol, Crocus Sativus Flower Extract.
